# Vertically aligned carbon nanotubes, MoS_2_–rGo based optoelectronic hybrids for NO_2_ gas sensing

**DOI:** 10.1038/s41598-020-68388-2

**Published:** 2020-07-09

**Authors:** Foad Ghasemi

**Affiliations:** Nanoscale Physics Device Lab (NPDL), Department of Physics, University of Kurdistan, Sanandaj, 66177-15175 Iran

**Keywords:** Materials science, Nanoscience and technology, Optics and photonics, Applied physics, Electronics, photonics and device physics

## Abstract

A simple method is developed through drop-casting techniques to assemble a molybdenum disulfide (MoS_2_)-reduced graphene oxide (rGO) hybrid on vertically aligned carbon nanotubes (VACNTs) to perform as an optoelectronic device for nitrogen dioxide (NO_2_) gas sensing at room temperature. The VACNT not only forms an ohmic contact with the hybrid material, but also yields a weak charge impurity scattering in the rGo layers across the channel. These features dramatically affect the optical response of the device to the light through which improve the photoresponsivity by up to 236% and the response time by up to 40% compared to the Au contacted device. Next, the fabricated MoS_2_–rGo/VACNTs device is employed as a resistor gas sensor for NO_2_ under in situ exposure to the light at room temperature. Under laser illumination, the sensor demonstrates a high sensitivity of ~ 41% at an inlet NO_2_ concentration of 100 ppm with a complete recovery time of ~ 150 s which shows comparable improvements relative to the sensor performance in dark condition.

## Introduction

Industrial development and economic expansion along with global population growth have dramatically increased supply and demand of commodities in the world^1^. These have caused many toxic gases such as nitride oxide (NO), nitrogen dioxide (NO_2_), ammonia (NH_3_), volatile organic compounds (VOCs), and formaldehyde to be released into the environment and threaten human health^2^. Because of the human lifestyle, the greatest risk is directly dominated by NO_2_ gas, which increases the chance of lung and respiratory diseases even at a low concentration^2^. Hence, it poses serious health problems in the human population and an argent monitoring of NO_2_ pollutants is highly in need of developing^2^. Therefore, efficient detection of the NO_2_ gas and development of the high performance semiconductor based sensors as promising candidates are crucial for protection of human health^3^. Atomic thickness and high surface-to-volume ratio of two-dimensional (2d) materials make them good candidates for gas sensing applications because their electronic transport properties are highly dependent on the surrounding atmosphere^4–6^. Despite the introduction of many new 2d materials, graphene as the first material discovered, is still used in many ongoing sensing studies thanks to its high electrical conductivity, large detection surface area, and high sensitivity potentials^7,8^. However, the desorption of the adsorbed molecules onto the surface of the graphene in the sensor channel does not occur easily resulting in a loss of sensitivity, poor response, and incomplete recovery of the sensor at room temperature^9^. Although, increasing the operating temperature up to 200 °C is associated with an improvement of the sensor performance, higher energy consumption and safety concerns limit their practical usages^7^. As an alternative suggestion, the required energy can be provided by light irradiation^8,9^. In fact, in situ exposure to light during gas detection process results in the photo-generated carriers within the channel of the optical-sensitive device. It can enhance the gas response of the sensor by two reasons: first, the photogenerated carriers provide the required energy for desorbing the ambient molecules (O_2_, and H_2_O) from the surface of the material. Hence, active sites can be released for the absorption of the analyte molecules. Second, the photoexcited carriers accelerate desorption process of the adsorbed molecules by releasing the occupied sites during recovery time^10^.

As a result, various optical active materials are integrated with graphene to fabricate high performance gas sensors. The most prominent candidates are the 2d materials^10,11^. Among various 2d materials^12,13^, transition metal dichalcogenides (TMDs), especially molybdenum disulfide (MoS_2_), has attracted significant interest because of its thickness-tunable bandgap, reasonable electron mobility, and low noise levels^14–16^.

Long et al. developed an MoS_2_/graphene sensor for NO_2_ detection at an operating temperature of 200 °C^17^. The sensitivity of sensor was reported about 15% at 3 ppm with recovery time of 1 min. Hong et al. fabricated a CVD growth MoS_2_/graphene hybrid with a sensitivity of 43% in response to 5 ppm NO_2_ concentration and fast response/recovery time of less than 1 s at working temperature of 200 °C^3^. A MoS_2_/graphene hetrostructure based NO_2_ sensor was introduced by Cho et al. with sensitivity range of 3 to 7% at 5 ppm gas concentration and recovery time of ~ 30 min at working temperature of 150 °C^18^. Deokar et al. presented a MoS_2_/CNTs hybrid with two-step operating processes: a detection step at room temperature and a recovery step at 100 °C. The sensitivity was measured to be 0.192% × ppm^−1^ at concentration range of 25–100 ppm^19^. Wang et al. took advantage of visible light illumination to detect formaldehyde gas by a MoS_2_/rGo hybrid gas sensor^20^. The sensor sensitivity was increased by 8 times at 10 ppm formaldehyde gas in comparison with the device in dark condition. Moreover, response time of the sensor was decreased from 79 s (in dark) to 17 s under light illumination. Kumar et al. fabricated a MoS_2_ based NO_2_ gas sensor that demonstrated a sensitivity 0f 17% at 5 ppm gas with recovery time of 350 s under UV illumination at room temperature^16^. An optoelectronic NO_2_ gas sensor was reported based on the single layer MoS_2_ layers with graphene contacts by Pham et al.^11^. Their results showed the device sensitivity of ~ 25% at 0.2 ppm NO_2_ gas with recovery time of about 200 s in situ exposure to red light at room temperature. However, in most reported works, the operating temperatures are above room temperature because they mostly showed poor responses and incomplete recovery times at near room temperature.

In this study, a MoS_2_–rGo hybrid is employed for the first time to detect NO_2_ gas based on the VACNTs electrical contacts. This structure was first proposed in our previous report^21^. Unlike previous work, here the rGo layer is suspended on VACNTs. For this purpose, Go solution was prepared in ethanol solvent and exposed to sonication process for a longer time to achieve small lateral size sheets. Surface tension of ethanol is lower than water and it is expected that it does not disrupt the arrangement of nanotubes during the drop casting process. Therefore, the small Go sheets, along with the low surface tension of the solvent, helped to place the layers suspended on the nanotube (Figure S1). The use of suspended rGo, in addition to improving its electrical properties, greatly increases the MoS_2_ optical response. In this work, the device performance is comprehensively evaluated and effect of electrical contacts and substrate on the electrical and optical properties of rGo–MoS_2_ hybrids are investigated. The results demonstrate an excellent optical improvement of the MoS_2_–rGo hybrid with VACNT contacts compared with the Au-contacted device. The optical responsivities of the device are investigated under different laser illuminations and in dark. In the case of VACNTs contacts, the photoresponsivity and rise time of 11.6 A/W and 0.55 s are obtained, respectively. Such a light-sensitive device can go one step further and be used to detect NO_2_ gas so that the process of gas detection takes place in situ exposure to the light illumination. Based on the results, the sensitivity of 41% and recovery time of ~ 150 s are measured that are comparable with the sensor performance in dark condition.

## Results and discussion

**Figure 1:**
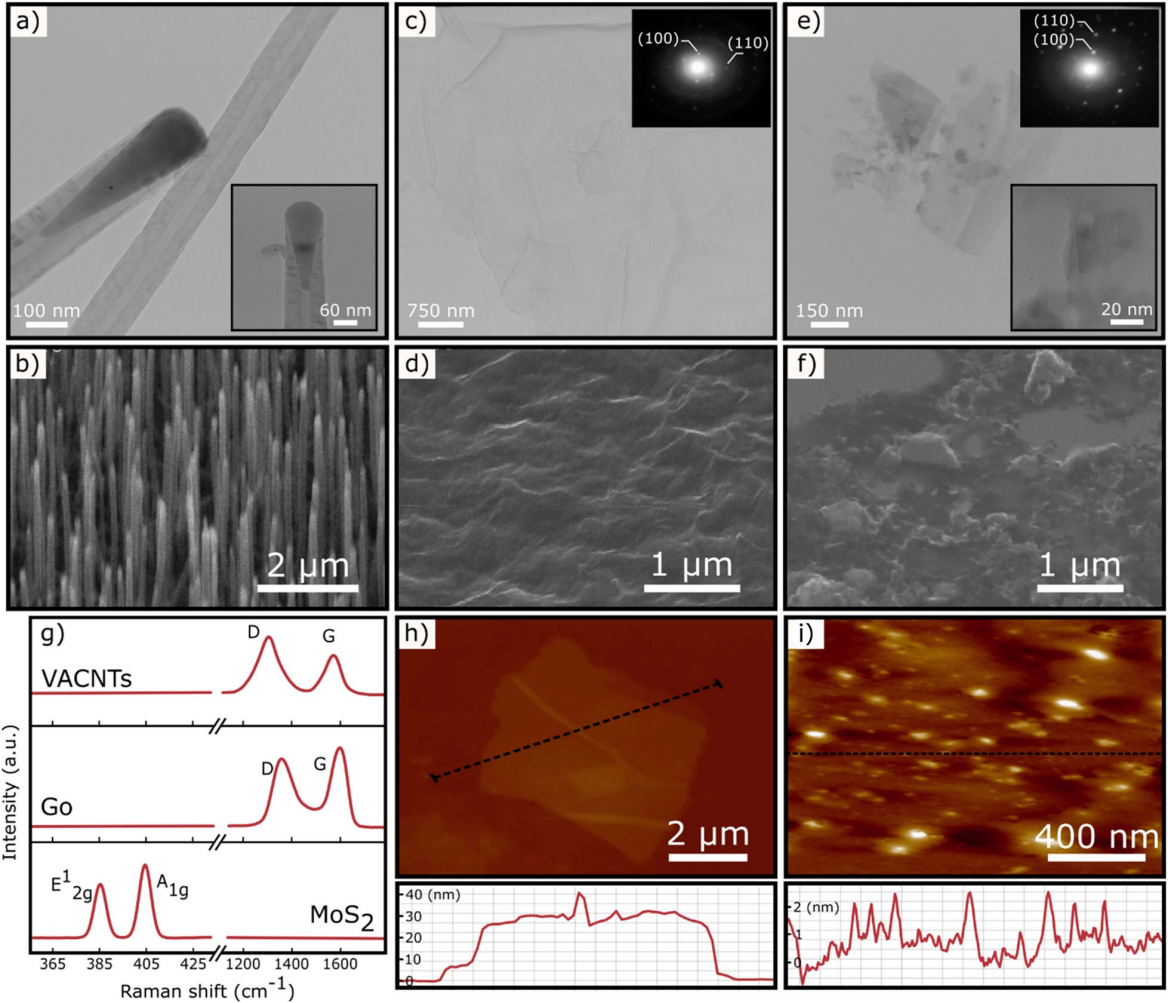
Material characterizations: (**a**) TEM image of VACNTs. Inset shows the high magnification image of VACNTs. (**b**) SEM image of VACNTs grown by the dc-PECVD system. (**c**) TEM image of Go layers and corresponding SAED pattern (inset). (**d**) SEM image of Go layer cast on a silicon substrate. (**e**) TEM image of MoS_2_ few layers along with high-resolution image and corresponding SAED pattern (insets). (**f**) SEM image of the few layers MoS_2_ cast on a silicon substrate. (**g**) Raman spectra of VACNTs, Go, and MoS_2_ samples. (**h**) AFM measurement of a Go layer with corresponding height profile. (**i**) AFM measurement of MoS_2_ nanosheets with the corresponding height profile.

Vertically aligned carbon nanotubes (VACNTs) were grown by a dc-PECVD system^21^ (see methods). Figure 1a exhibits the transmission electron microscopy (TEM) image of the grown VACNTs. Based on it, the approximated diameter is recorded to be in range of 30–100 nm. The scanning electron microscopy (SEM) image of VACNTs also demonstrates the dense growth of nanotubes with a vertically aligned arrangement where the lengths of the nanotubes are measured to be around 1–2 µm (Fig. 1b). In the growth process of VACNTs, Ni film acts as a catalyst which remains on the top of the nanotubes upon completion of the growth (inset of Fig. 1b). However, catalyst particle is surrounded by a thin carbon layer and buried inside nanotubes^21^. GO dispersion was synthesized by a modified Hummer’s method^22^ (see methods). The TEM image of the Go few layers is presented in Fig. 1c where its transparency and corrugated structure prove the suitable exfoliation of initial flakes. Moreover, the selected area electron diffraction (SAED) pattern shows the hexagonal crystalline structure of the Go sheet^22^. The fuzzy rings in the SAED pattern may be because of the presence of oxygen containing functional groups in the Go surface^22^. SEM image of the Go sheet indicates the wrinkle morphology of layers (Fig. 1d) that are well distributed across the bare silicon substrate. MoS_2_ dispersion was prepared by a solvent based liquid-phase exfoliation method^23^ (see methods). TEM image of the MoS_2_ nanosheets is exhibited in Fig. 1e through which the layered structure of the sheets is observable. Moreover, the insets show a high-resolution TEM image and a SAED pattern of the MoS_2_ sheet. The former implies the electron transparency of the layers and the latter confirms the crystalline structure of the sheets^23^. The morphology of the deposited MoS_2_ nanosheets is also presented in the SEM image in Fig. 1f. For this purpose, a few droplets of MoS_2_ solution were drop cast onto a silicon wafer piece and then vacuum-dried overnight at room temperature. This low temperature evaporation process allows the nanosheets to be uniformly distributed across the substrate (Figure S2).

Raman spectroscopy and atomic force microscopy (AFM) analyses were also employed to study the final product quality of the VACNTs, Go and MoS_2_ samples (part g–k of the Fig. 1). In the case of VACNTs, the grown sample (on SiO_2_/Si substrate) was directly placed in the Raman system, while for two other samples, several drops of the prepared solutions were cast onto the SiO_2_/Si substrates and vacuum-dried at 70 ˚C. The Raman spectrum of the VACNTs demonstrates two characteristic peaks located at ~ 1,305 cm^−1^ (D band) and 1,580 cm^−1^ (G band)^21^. For Go few layers, these peaks are around ~ 1,360 cm^−1^ and 1595 cm^−1^, respectively^22^. E^1^_2g_ and A_1g_ modes of the MoS_2_ flakes were observed near 385 cm^−1^ and 403 cm^−1^ corresponding to the in-plane and out-plane phonon oscillation modes, respectively^23^. AFM measurement of a Go flake and corresponding height profile are presented in Fig. 1h. According to the height profile, the thickness of flake is measured to be ~ 30 nm. Moreover, the AFM image of the MoS_2_ sample shows the well deposition of the few-layered MoS_2_ nanosheets across the substrate. It should be noted that the Go and MoS_2_ monolayers have both about 0.8 nm in thickness^22,23^.

**Figure 2:**
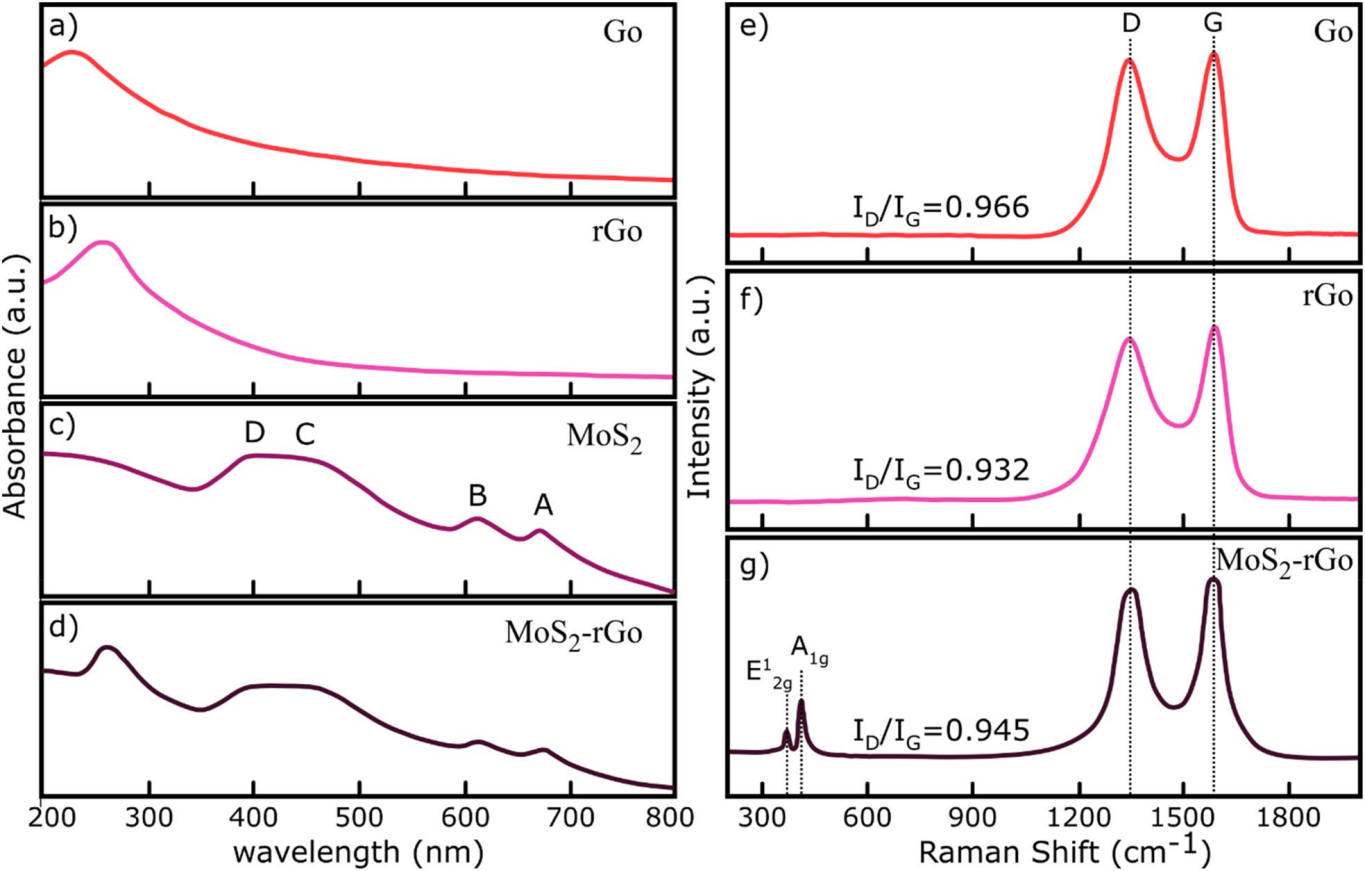
UV–visible and Raman characterizations. (**a**) UV–visible spectrum of Go. (**b**) UV–visible spectrum of rGo. (**c)** UV–visible spectrum of MoS_2_ nanosheets. (**d**) UV–visible spectrum of MoS_2_–rGo hybrid. (**e**) Raman spectrum of Go few layers. (**f**) Raman spectrum of rGo few layers responds to part e. (**g**) Raman spectrum of MoS_2_–rGo hybrid.

As shown in Fig. 2a–d, the UV–visible absorption spectra of the Go, rGo, MoS_2_, and MoS_2_–rGo samples are represented. In the case of Go (Fig. 2a), a sharp absorption peak is observed at ~ 230 nm related to the π → π* transitions of the C–C bonds^24^. A small shoulder peak around 325 nm is also attributed to the n → π* transitions of the C = O bonds^24^. For rGo (Fig. 2b), the absorption peak is red shifted to the ~ 260 nm because of removal of the oxygen containing functional groups and corresponding increase in the electron concentrations^25^. The absorption spectrum of the MoS_2_ sample exhibits characteristic peaks at 390 nm, 460 nm, 612 nm, and 675 nm corresponding to the D, C, B, and A excitonic transitions^23,26^ (Fig. 2c). The two former exciton peaks (D and C) corresponds to the direct transitions to the conduction bands from the deeper valence bands. A and B exciton peaks attribute to the direct transition at the K point of the Brillouin zone^27^. The presence of these two peaks not only indicates the high quality product of the MoS_2_ multilayers but also confirms the existence of the MoS_2_ monolayers in the samples^23^. For the MoS_2_–rGo sample, in addition to the MoS_2_ peaks, a strong peak is observed at 260 nm originating from the rGo contribution. Such a spectrum clearly shows the well formation of the hybridized MoS_2_–rGo structure. Therefore, the optical absorption of the obtained composite is ranged from 200 to 800 nm. However, a dominant absorption is observed for energies less than 500 nm.

The Raman spectra of the Go, rGo, and MoS_2_–rGo samples are presented in Fig. 2e–g. Two signal peaks at about 1,590 cm^−1^ and 1,350 cm^−1^ refer to D and G band vibration modes of Go layers, respectively^22^ (Fig. 2e). From an electrical point of view, Go is known as an insulator and its electrical conductivity needs to be improved by elimination of the oxygen functional groups in its surface. A variety of methods are introduced to reduce the Go layer such as thermal reduction, UV irradiation, and chemical reduction techniques^22^. In the thermal reduction technique, the Go sample is heated up in a gas atmosphere^22^. During the process, functional groups receive the energy needed to break the bonds separated from the Go flakes and leave the surface. The thermal reduction of the Go flake is performed as follows: a few droplets of a Go solution were cast onto the SiO_2_/Si substrate. After drying at room temperature, it was placed in a quartz furnace and vacuumed. The temperature was then risen to 600 °C in the presence of argon gas (12 sccm and 1 torr), remained at the final temperature for 60 min, and finally cooled to room temperature under the same condition. Based on the Fig. 2f, the Raman spectrum of rGo also has two characteristic peaks at 1,590 cm^−1^ and 1,350 cm^−1^ indicating G and D oscillation modes, respectively^21^. By measuring the I_D_/I_G_ ratio in both Go and rGo Raman spectra, it is observed that the ratio decreases from 0.966 for Go to 0.932 for rGo as a result of well elimination of the functional groups on the Go surfaces^22^. Subsequently, a few droplets of a MoS_2_ solution was cast onto the prepared rGo layers and vacuum-dried at 110 °C for 2 h. In the Raman spectra of the hybrid structure, the peaks associated with rGo and few-layered MoS_2_ are clearly observed (Fig. 2g). The two peaks around 385 cm^−1^ and 405 cm^−1^ reveal the E^1^_2g_ and A_1g_ oscillation modes of the MoS_2_ multilayers^23^, and the other two peaks at 1,350 cm^−1^ and 1,590 cm^−1^ are attributed to the D and G phonon vibrational modes of the rGo layers, respectively. By measuring the peak intensity ratio of D to G bands in the resulting spectrum it is found that the ratio increases to 0.945, which may be originated from interaction between few the layered MoS_2_ and the rGo sheets which implies a good hybrid structure formation^22^.

**Figure 3:**
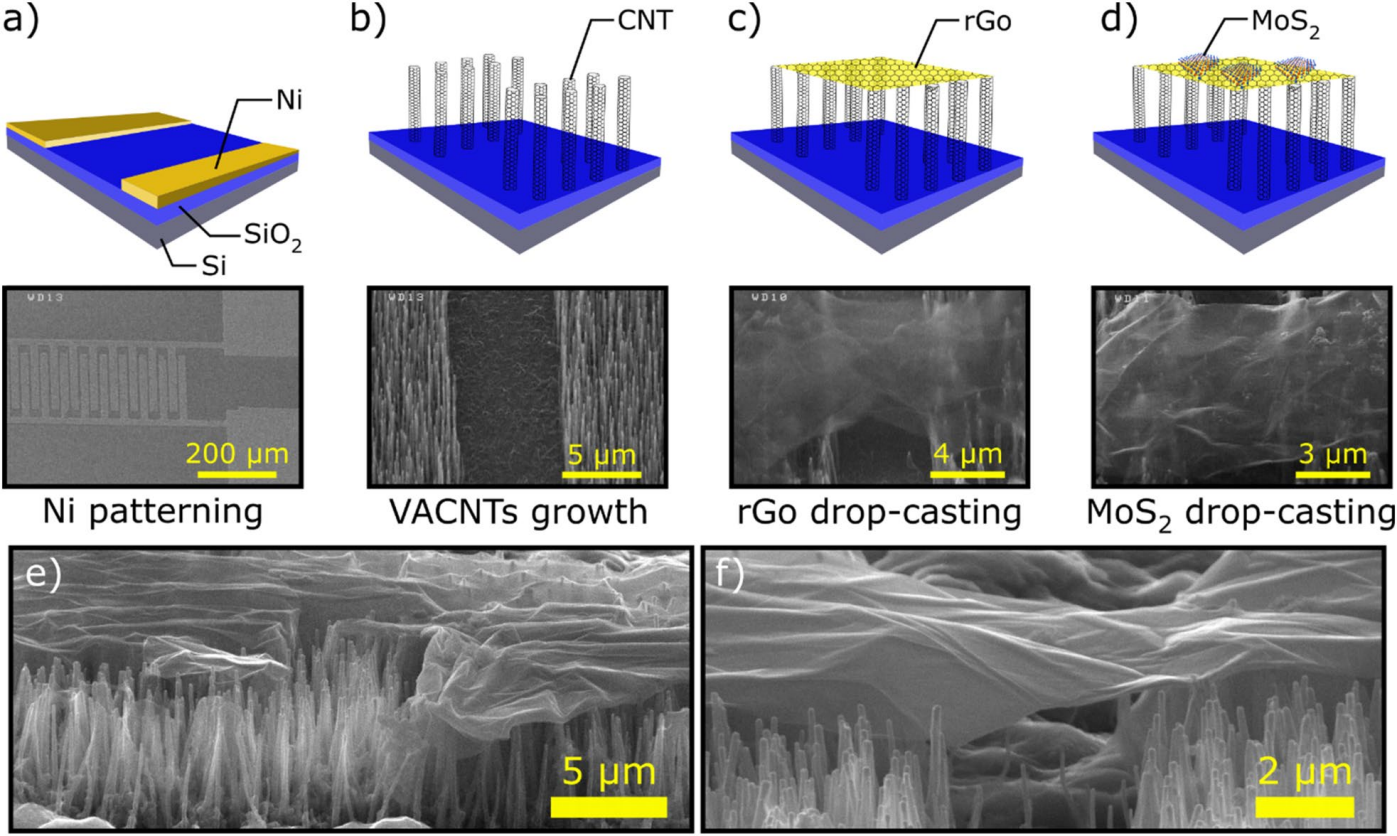
Schematic illustrations of the device fabrication steps: (**a**) photolithography patterning of the Ni thin film. (**b**) The dc-PECVD growth of the vertically aligned CNTs. (**c**) Drop-casting of the Go layers and reducing into the rGo layers. (**d**) Drop-casting of the MoS_2_ few layers on the rGo/VACNTs electrode. (**e**) Cross-sectional image of rGo on VACNTs. (**f**) Cross-sectional image of rGo layer over the VACNTs source/drain electrodes.

**Figure 4:**
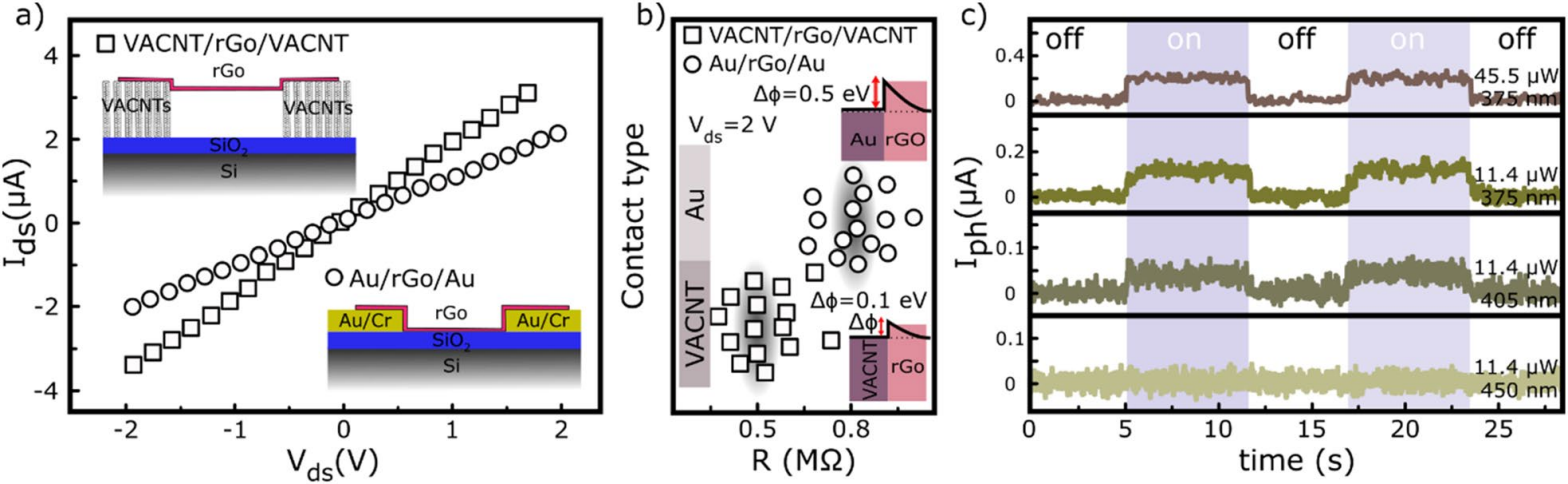
Electrical characterizations: (**a**) I–V characteristic of the VACNTs- and Au- contacted rGo devices. (**b**) Resistance distribution of both devices and their corresponding Schottky barrier heights. (**c**) Optical response of the rGo/VACNTs device under 450, 405, and 375 nm laser illuminations.

Figure 3 schematically illustrates the fabrication steps of the device with corresponding SEM images. Cross-sectional images of the rGo layer over VACNTs are also presented in part e and f of Fig. 3. Before evaluation the performance of MoS_2_–rGo/VACNTs device in response to NO_2_ gas, the effect of the VACNTs contacts on the optoelectronic performance of the device was investigated compared to the Au contacts. For this reason, two types of devices were fabricated. The first is Au and the second VACNTs electrical contacts. Figure 4a shows the I–V characteristics of the two types of devices. Accordingly, the higher current passed through VACNTs contacts than Au (100 nm) contacts. For both types, 15 devices were prepared and their electrical resistivities were measured (Fig. 4b). In most cases, less electrical resistance was obtained for VACNTs padded device. There are two reasons to justify this behavior: First, the VACNT-rGo junction has a lower Schottky barrier height (SBH) than Au-rGo junction^21,28^. In fact, in the case of VACNT-rGo, both structures are composed of the carbon atoms and allow carriers to pass easily between them. The work function of rGo is ~ 4.9 eV and the work function of carbon nanotube is about 5 eV, while the Au work function is 5.4 eV^21^. Therefore, the SBH at the VACNTs-rGo interface is expected to be about ~ 0.1 eV, while for Au-rGo interface is about ~ 0.5 eV (Insets of Fig. 4b). Moreover, due to the large height of the VACNTs (about one microns), the rGo layers are completely suspended across the channel between source/drain electrodes as the SEM images shows. It reduces the scattering effect of the surface polar phonons (SPP) originating from charge impurity in channel/substrate interface and thus increases the carrier mobility within the channel^29^. Therefore, the VACNT-rGo junction has a superior advantage over the Au-rGo junction in the final electrical performance of the device.

In the case of VACNT junction, the optical response of the device is evaluated at different excitation wavelengths at a biasing voltage of 2 V and a power illumination of 11.4 µW (Fig. 4c). Based on the results, a relatively poor optical response is observed only at excitation wavelengths of 405 and 375 nm. At an incident power of 11.4 µW, the photocurrents are increased by 40 and 110 nA for the wavelengths of 405 and 375 nm, respectively. By increasing the light intensity to 45.5 µW, the photocurrent is also increased by 200 nA. However, no optical response was observed for the lower radiation wavelengths. This result is also consistent with the absorption spectrum of the rGo because it has more optical absorption in the ultraviolet region.

MoS_2_ nanosheets are used to enhance the optical response of the device. For this purpose, a few droplets of MoS_2_ solution are drop cast onto the rGo/VACNTs electrode and after drying it, the final device is annealed at 110 °C for 2 h (Fig. 3d).

**Figure 5:**
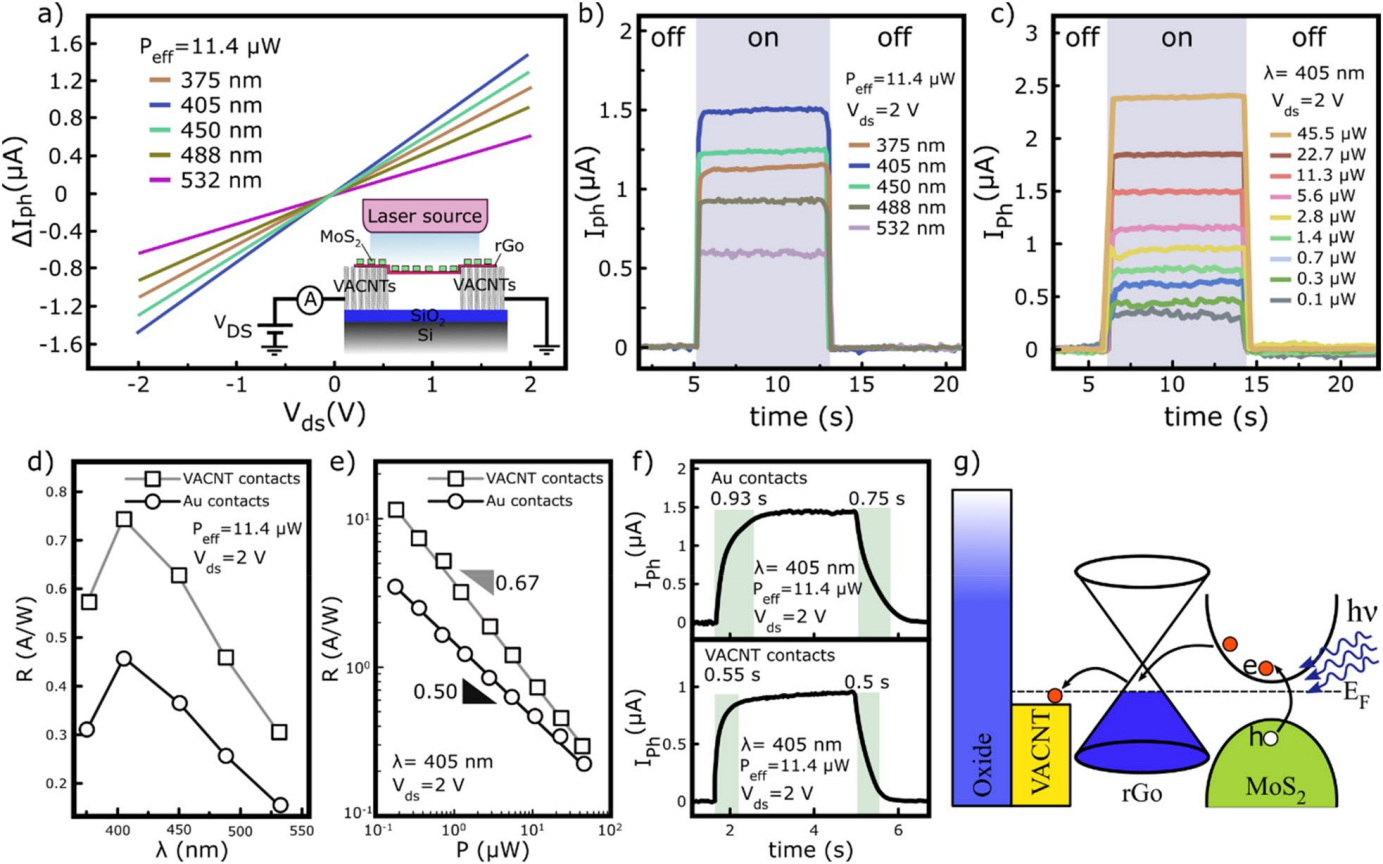
Optoelectronic characterization of the fabricated devices. (**a**) The photogenerated current in MoS_2_–rGo/VACNTs photodetector under various laser irradiations with same intensity of 11.4 μW. Inset shows the corresponding electrical measurement circuit. (**b**) The photoswitching measurements of the MoS_2_–rGo/VACNTs photodetector under various laser illuminations at an optical power of 11.4 μW and a biasing voltage of 2 V. (**c**) The photoswitching measurements of the MoS_2_–rGo/VACNTs photodetector under 405 nm laser illumination at different optical powers and a biasing voltage of 2 V. (**d**) The photoresponsivity verses excitation wavelengths for the VACNT- and Au- contacted devices at an optical power of 11.4 μW and 2 v bias voltage. (**e**) The Photoresponsivity verses incident light intensities for the VACNT- and Au- contacted devices under laser illumination of 405 nm and 2 v bias voltage. (**f**) Time resolved measurements of the VACNT and Au contacted devices under 405 nm laser irradiation at a power intensity of 11.4 μW and a biasing voltage of 2 V. (**g**) Energy band diagram of the MoS_2_–rGo/VACNTs photodetector under light illumination.

After fabrication of the MoS_2_–rGo/VACNTs device, electrical behavior and optical response of the device are evaluated under different laser wavelengths and power intensities at room temperature in ambient air. Figure 5a demonstrates the generated photocurrents in the device under different wavelength illuminations at an incident power of 11.4 μW. The inset also shows the electrical measurement circuit of the device. It is observed that the highest photocurrent obtains at a laser excitation wavelength of 405 nm. After that, the next highest photocurrents occur for the 450 and 375 nm wavelengths, respectively. Although the energy of the incident photon in the latter laser (375 nm) is greater than the first two wavelengths, it results in less optical response in the device. This behavior is consistent with the absorption spectrum of the MoS_2_–rGo hybrids as in the 375 nm range, a less optical absorption is observed in the composite structure (Fig. 2d). By increasing the wavelength of the incident photons to 488 and 532 nm, less photocurrents are generated in the device. It is worth noting that the photo response performance of the device is well consistent with the absorption spectrum of the MoS_2_–rGo hybrids. Figure 5b compares the photocurrents generated for different laser wavelengths at a constant power intensity of 11.4 µW and a biasing voltage of 1 V. Accordingly, photocurrents of ~ 1.50, 1.27, 1.15, 0.93 and 0.61 μA are measured for the wavelengths of 405, 450, 375, 488 and 532 nm, respectively. Figure 5c shows the photocurrent measurements under 405 nm laser illumination at 2 V biasing voltage for different power intensities. Based on it, an increase in the incident power results in more electron/hole pairs generation and subsequently leads to the photocurrent enhancement^30^. In details, the photocurrents of ~ 2.40, 1.85, 1.51, 1.15, 0.95, 0.77, 0.65, 0.46, and 0.37 µA are obtained for the effective power intensities of 45.56, 11.78, 11.40, 5.69, 2.84, 1.42, 0.71, 0.35, and 0.17 µW, respectively. In addition to the photocurrent, the photoresponsivity (R) of the both devices with VACNT and Au junctions is measured in terms of wavelength and incident power. The R is calculated based on the following Eq. (1)^30^:

1 $$ R = \frac{{I_{ph} }}{{P\frac{{A_{d} }}{{A_{l} }}}} $$

where I_ph_, P, A_d_, and A_l_ are the photocurrent, optical power, active area of the device and illumination area of the laser source, respectively. In all measurements, a higher photoresponsivity is measured for the VACNTs contact, because of the lower SBH at the junctions, which facilitated the extraction/injection of electron–hole carrier pairs within the channel. In both devices, the highest photoresponsivity occurred at 405 nm laser illumination for the same optical power of 11.4 µW. Accordingly, the photoresponsivities of ~ 0.74 A/W and ~ 0.45 A/W are obtained for the devices with VACNTs and Au junctions, respectively. Figure 5d shows the photoresponsivity characteristic in terms of wavelengths for both types of electrical contacts. Based on it, with increasing the wavelength of the laser source, the photoresponsivity decreases in both types of junctions. The peak observed in the photoresponsivity behavior exhibits the highest sensitivity at 405 nm because of the presence of C and D exciton transitions of MoS_2_ nanosheets. Moreover, in the case of 405 nm laser illumination, the photoresponsivity is measured as a function of the optical power for both devices at 2 V biasing voltage (Fig. 5e). The R values decrease with increasing incident powers. This behavior can be justified as follows: increasing the optical power generates more electron–hole pairs, which increases the probability of carrier being captured in traps and hence decreases the R^30^. For Au contacted device, a lower photoresponsivity value is measured than the VACNTs contacted device, indicating the importance of the SBH. The photoresponsivities of 11.67 A/W and 3.47 A/W are obtained for the VACNTs and Au junctions at a 0.17 µW incident power of 405 nm radiation, respectively. The photoresponsivity can be fitted as a function of optical power by a power law, R ∝ P^β−1^ where β is a dimensionless parameter relating to the recombination kinetics of the photoexcited carriers^21,30^. In the absence of trap states, β takes 1 value and it gets close to 0 in the presence of trap states^31^. Based on the power law, β values of 0.33 and 0.50 are obtained for VACNT- and Au- padded devices, respectively. The difference in the fitted value of β may be related to the surface polar phonon (PPS) contribution because of the strong interaction between the photogenerated carriers and the charge impurity scattering from the substrate^29^. The large height of the VACNTs results in the suspended rGo layers across the channel, which reduces its PPS interaction with the substrate. Therefore, the decrease in the phonon interactions and the low SBH Schottky barrier in the VACNT contacted device enhance the carrier mobility and their chances of reaching the junctions in the applied bias field in comparison to the Au-contacted device^29^. The fitted parameter of β in the power law illustrates this difference well.

The time response of the fabricated devices are presented in Fig. 5f under 405 nm laser illumination with 11.4 μW optical power and a 2 V biasing voltage. Rise time is defined as the time duration taken by photocurrent to reach 90% from 10% and fall time is known as the time duration to decay photocurrent from 90 to 10%. According to the results, rise time and fall time of 0.55 s and 0.50 s are measured for VACNT contacted device, but these values increased to 0.93 s and 0.75 s for Au contacted device. A low SBH and an absence of charge impurity scattering are the important factor in the rapid optical response of VACNT- relative to Au- contacted devices^29^. Figure 5g shows the energy band diagram of the MoS_2_–rGo device with VACNT junctions. Under light irradiation, the electron/hole pairs are generated in MoS_2_ nanosheets. The excited electrons are transferred to the rGo layer while the holes are trapped in the MoS_2_ nanosheets. In rGo, the high electron mobility and the long trapping of hole carriers cause the electrons to circulate in the channel before being recombined^21^. The low electron affinity of MoS_2_ (~ 4.0 eV) and the high work function of rGo (~ 4.9 eV) allow the electron to be easily transferred from MoS_2_ to rGo^21^. Moreover, the ohmic resistance between rGo and VACNT junction accelerates the extraction/injection of carriers in the channel^21,29^.

**Figure 6:**
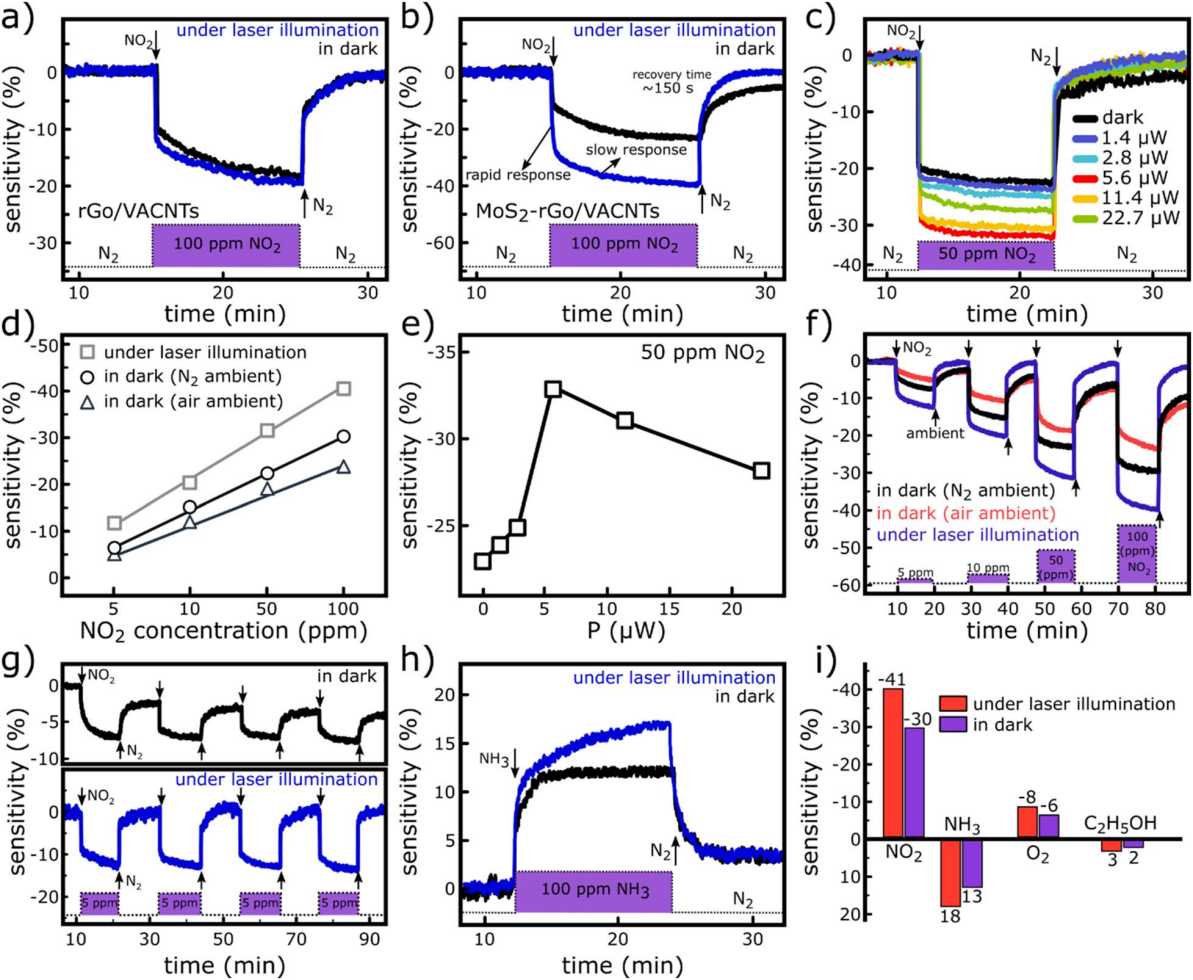
Gas sensing performance of the fabricated sensor. (**a**) Sensitivity of the rGo/VACNTs sensor to 100 ppm NO_2_ gas under laser illumination and in dark conditions. (**b**) Sensitivity of the MoS_2_–rGo/VACNTs sensor to 100 ppm NO_2_ gas under laser illumination and in dark conditions. (**c)** Sensitivity of the MoS_2_–rGo/VACNTs sensor as a function of the laser power intensity to 50 ppm NO_2_ gas. (**d**) Sensitivity as function of NO_2_ gas concentrations in situ laser illumination and in dark. In the case of dark condition, the N_2_ and air ambients are compared. (**e)** The sensitivity dependency of MoS_2_–rGo/VACNTs sensor on the incident light intensity to 50 ppm NO_2_ gas. (**f**) Sensing performance of the sensor to different NO_2_ gas concentrations (5, 10, 50, and 100 ppm) in dark (N_2_ and air ambients) and under laser illumination. (**g)** Cyclic measurements of the sensor in response to the 5 ppm NO_2_ gas in dark and under laser irradiation. (**h)** The response of the MoS_2_–rGo/VACNTs sensor to 100 ppm NH_3_ gas in dark and under laser illumination. (**i**) Comparison of the sensor selectivity to NO_2_, NH_3_, O_2_, and C_2_H_5_OH inlet gases with 100 ppm concentration in dark (N_2_ ambient) and under laser illumination at room temperature.

Based on the results of the previous section, it was found that the optical response capability of the VACNT contacted device is significantly better than the Au padded device. Therefore, this device was used to investigate the NO_2_ gas sensing properties. Figure 6 compares the sensor performance in response to NO_2_ gas in dark and under laser irradiation at a biasing voltage of 2 V. The sensitivity of device was calculated based on the Eq. (2)^32^:

2 $$ S = \frac{{R_{{NO_{2} }} - R_{{N_{2} }} }}{{R_{{N_{2} }} }} \times 100\% $$

where $R_{{NO_{2} }}$ and $R_{{N_{2} }}$ are the sensor resistance in the presence of NO_2_ and N_2_ gases, respectively. Figure 6a shows the performance of the rGo/VACNTs sensor in response to 100 ppm NO_2_ gas in dark and under laser illumination (405 nm, and 5.6 μW power intensity). It is observed that the sensitivity of the sensor has not changed significantly under laser illumination (-21%) compared with the dark condition (-−19%). Figure 6b compares the MoS_2_–rGo/VACNTs sensor performance in response to NO_2_ gas in dark and under 405 nm laser irradiation with 5.6 μW optical power at a biasing voltage of 2 V. The overall behavior of MoS_2_–rGo/VACNTs device shows a p-type semiconductor because rGo layers are the dominant structure contribute in both composition and total electrical conductance of the sensor^33^. Therefore, upon exposure to NO_2_ gas, the device resistance decreases and the sensitivity gets a negative value. For an inlet NO_2_ concentration of 100 ppm, the device demonstrates a sensitivity of ~ −31% with recovery time more than 10 min in dark condition whereas the sensitivity increases to ~ -41% and the recovery time decreases to ~ 150 s under laser illumination at room temperature (Fig. 6b). These results suggest that MoS_2_ nanosheets in addition to providing active sites for absorption of the analytical molecule, enhance significantly the sensing performance of device by making it optically active. Light radiation is associated with the generation of the electrons/holes pairs. The photogenerated carriers release sites occupied by ambient molecules (such as O_2_ and H_2_O molecules) on the flakes surface and make them active sites for the adsorption of NO_2_ molecules^10^. They also provide the activation energy needed to desorb the trapped molecules during the recovery time, thereby improving the sensitivity and performance of the device^10,11,15,16^. Two type of active sites contribute in the absorption of NO_2_ molecules on the MoS_2_–rGo flakes including low energy adsorption sites (sp^2^ bonded carbon) and high energy adsorption sites^17^ (defects, sulfur/oxygen vacancies). The former is responsible for the rapid response of the sensor that interacts poorly with the guest molecules whereas the slow response occurs through the strong interaction of the molecules with the energetic sites^17^. Although the presence of these high-energy sites increases the sensitivity, they lead to a slow recovery time, which makes the sensors not perform optimally at room temperature unless irradiated photons supply the activation energy needed to desorb the NO_2_ molecules. Figure 6c exhibits the response of the sensor to NO_2_ gas in term of the incident light intensity. It is noteworthy that with increasing the light intensity, the sensitivity also increases but for incident powers of more than 5.6 μW a significant decrease is observed. Incident light generates electrons/holes pairs, however, at higher intensities, the traps capture the photogenerated carriers and reduce the photoresponsivity, thereby reducing the gaseous sensitivity^16^. Therefore, the maximum sensitivity of sensor is observed at the light intensity of 5.6 μW. The variation of sensitivity versus the incident optical power at the NO_2_ concentration of 50 ppm is also displayed in Fig. 6d. The sensitivity of the sensor as a function of the inlet NO_2_ concentration is presented in Fig. 6e in dark (air and N_2_ ambients) and under light exposure (405 nm wavelength, 5.6 μW incident power, and 2 V biasing voltage). At all concentrations, the higher sensitivities are obtained for the sensor exposed to the light. For the inlet NO_2_ concentrations of 5, 10, 50, and 100 ppm the sensitivities of −7, −15, −23, and −30% were obtained in dark (in N_2_ ambient) and these values increased to −13, −21, −33 and −41%, under 405 nm laser illumination, respectively. Furthermore, the lower values of the −5, −12, −18, and −23% were obtained for the sensor’ sensitivity in air ambient in dark compared with the N_2_ conditions. The decrease of the sensor performance in the air ambient can be due to the occupation of the flakes active sites by ambient molecules, which reduces the absorption possibility of the analytical molecules. The dynamic performance of the MoS_2_–rGo/VACNTs sensor is also evaluated toward various inlet NO_2_ concentrations from 5 to 100 ppm in dark (air and N_2_ ambients) and under laser illumination at room temperature (Fig. 6f). In the case of dark condition, the sensor shows a poor recovery at room temperature where the slower response is more noticeable in ambient air conditions. Moreover, the cyclic sensing performance of the device is measured in response to 5 ppm NO_2_ concentration in dark and under light irradiation at room temperature in N_2_ ambient (Fig. 6g). Under laser illumination condition, a complete recovery with fast response of ~ 150 s is observed which leads to the excellent reversibility of the device at room temperature.

Figure 6h also shows the sensitivity of the sensor to 100 ppm NH_3_ concentration in dark and under laser illumination in N_2_ ambient at room temperature. Based on it, the sensitivity is measured to be around 18% under laser illumination and about 13% in dark condition. Figure 6k compares the response values of the sensor to different gases (NO_2_, NH_3_, O_2_, and C_2_H_5_OH) in dark and under laser illumination in N_2_ ambient at room temperature. Under laser illumination, the response values of −41, 18, −8, and 3% were obtained for NO_2_, NH_3_, O_2_, and C_2_H_5_OH gases at constant concentration of 100 ppm while these values were decreased to −30, 13, −6, and 2% in dark condition, respectively. Accordingly, the sensor demonstrates an excellent selectivity to NO_2_ gas in comparison to the other gas species. The positive and negative values of the sensitivity indicate a decrease and increase in the resistance of the sensor upon gas exposure, respectively.

The total resistance of the device divides into three parts^18^:

3 $$ {\text{R}}_{{{\text{total}}}} = {\text{R}}_{{{\text{channel}}}} + {\text{R}}_{{{\text{contact}}}} + {\text{R}}_{{{\text{electrode}}}} $$

The MoS_2_–rGo hybrid is responsible for R_channel_, which is highly depended on the inlet NO_2_ concentration and decreases with the presence of gas. R_contact_ is related to the SBH in the interface of rGo and VACNTs. Thanks to the carbon structure of both materials and the matched work functions, a low SBH appears in their interfaces, leading to an ohmic contact and easy circulation of carriers within the channel. This resistance depends on the nature of the two structures which after band alignment, it does not significantly affect by the inlet gas. The third part, R_electrode_, is associated with the resistance of the VACNTs. The presence of sp^2^ carbon bonds in the VACNTs makes them sensitive to the environment, especially NO_2_ molecules^34^. Therefore, the R_electrode_ can play a significant role in determining the overall value of R_total_. This can affect the final performance of the sensor especially here that the device would be favorable to operate at room temperature. To overcome this issue, after dc-PECVD growth of VACNTs, a post treatment process is applied by an irradiation of the nanotubes to the H_2_ plasma (1.5 W/cm^2^, 2 min, and 700 ˚C). The treatment step considerably decreases the reactive sp^2^ sites on VACNTs and make them hydrogenated and non-reactive against NO_2_ molecules^35^. Therefore, channel resistance is the only part of the R_total_ affected by the inlet gas, and other components only indirectly improve the sensitivity and overall performance of the sensor.

The mechanism of the fabricated MoS_2_–rGo/VACNTs sensor in response to the NO_2_ gas can be described as follows: both rGo and MoS_2_ flakes have active sites for absorbing ambient NO_2_, O_2_ and H_2_O molecules. In the thermally reduced GO, the active sites originate from defects and oxygen vacancies whereas in the MoS_2_ flakes edge states and sulfur vacancies act as active sites to accept guest molecules^17^. In NO_2_ molecule, the unpaired electron of the nitrogen atom makes the molecule behave as an electron acceptor. Moreover, rGo behaves as a p-type semiconductor and MoS_2_ known as an n-type semiconductor. It was found that the MoS_2_/rGo hybrid has a p-type behavior which may be because of the dominance of rGo to MoS_2_ in the hybrid structure and the dominant participation of rGo in the overall conductivity of the sensor^33^. During exposure of the sensor to NO_2_ gas, the gas molecules are absorbed by the active sites on the rGo and MoS_2_ flakes. At the edges of MoS_2_ nanoflakes, there are a lot of bridging sulfur atoms that provide extra active sites for absorbing the NO_2_ molecules^17^. This makes MoS_2_ more sensitive to NO_2_ molecules and absorbs more molecules than rGo. As a result, NO_2_ molecules mainly capture electrons from MoS_2_ nanoflakes causing an electron-depleted structure. The high electron mobility of rGo derive electrons to the less conductive MoS_2_ flakes. Therefore, the concentration of minority carrier (electron) significantly decreases in rGo and less electrons can reach the contacts and subsequently the device resistance decreases in the presence of NO_2_ gas^10^. Under laser illumination, many electron/hole pairs are generated which provide activation energy for desorption of adsorbed O_2_, H_2_O and NO_2_ molecules from both rGo and MoS_2_ surfaces. In addition to preventing sensor saturation, the photogenerated carriers provide further active sites for NO_2_ molecules by removing O_2_ and H_2_O molecules, leading to an increase of the device sensitivity. As a result, the recovery time of sensor can be improved and device acts as a rapid sensor even at room temperature^10^. The Eqs. (4) and (5) summarized the above discussion:

4 $$ \left( {H_{2} O^{ - } /O_{2}^{ - } - active site} \right) + hole + electron \rightleftharpoons active site + electron + \uparrow O_{2} /H_{2} O $$

5 $$ \left( {NO_{2} + active site} \right) + electron \rightleftharpoons \left( {NO_{2}^{ - } - active site} \right) $$

The fabricated MoS_2_–rGo/VACNTs sensor operates at room temperature with a final sensitivity of ~ −41% under 100 ppm NO_2_ concentration with a recovery time of ~ 150 s under 405 nm laser illumination. Table 1 summarizes a comparison between various fabricated NO_2_ sensors based on the MoS_2_ and rGo materials. It can be seen that the presented sensor shows a superior performance compared with other sensor importantly at working temperate of room.

**Table 1:** Comparison in sensor performance of the MoS_2_–rGo/VACNTs sensor with the devices in the literatures.

Material	Working temperature	NO_2_ concentration (ppm)	Response	Recovery time	Refs.
MoS_2_	RT (UV)	5	17%	350 s	^16^
MoS_2_	60 ˚C	5	17%	172 s	^15^
MoS_2_	200 ˚C	5	23%	107 s	^36^
Graphene–MoS_2_	150 ˚C	5	7%	> 10 min	^18^
Graphene–WS_2_	RT	2	7%	300 s	^32^
MoS_2_–Graphene	200 ˚C	3	15%	< 1 min	^17^
MoS_2_–Graphene	200 ˚C	5	43%	0.9 s	^3^
rGo-SnS_2_	150 ˚C	5	30%	48 s	^37^
MoS_2_–rGo	90 ˚C	5	25%	~ 300 s	^38^
MoS_2_-PbS QDs	RT	5	5%	62 s	^39^
MoS_2_–rGo	RT (red light)	0.2	25%	~ 200 s	^11^
MoS_2_-CNTs	100 ˚C	50	15%	> 300 s	^19^
WS_2_–rGo	50 ˚C (UV)	1	70%	18 min	^10^
MoS_2_–rGo	60 ˚C	6	55%	> 500 s	^33^
MoS_2_–rGo/VACNTs	RT (UV)	5	13%	~ 150 s	Here

## Conclusion

In summary, a novel method is employed to fabricate MoS_2_–rGo photodetector with VACNT electrical contacts. The electrical and optical properties of the prepared device was evaluated and compared with the Au padded MoS_2_–rGo device. Because of the low Schottky barrier height at the contact interface and decrease of the surface polar phonon scattering across the channel, the VACNT contacted device shows a superior performance with photoresponsivity of 11.6 A/W and ultrafast rise/fall times of 0.93/0.75 s, respectively. The fabricated MoS_2_–rGo/VACNTs device was then developed to detect the various concentrations of NO_2_ gas at room temperature. The sensor demonstrates an ultrafast response to NO_2_ gas with an excellent sensitivity of ~ −0.41% at 100 ppm NO_2_ and completed recovery time of ~ 150 s during sensing in-situ laser illumination compared with the sensing process in dark condition.

## Methods

### Materials

Graphite powder (purity > 99.9%, Fluka), hydrogen chloride (Sigma Aldrich), sodium nitride (Sigma Aldrich), hydrogen peroxide (Sigma Aldrich), sulfuric acid (Sigma Aldrich), potassium permanganate (Sigma Aldrich) and ethanol (Merck) were employed to synthesize graphene oxide few layers. A commercial MoS_2_ powder (purity > 99.0%, Sigma Aldrich) and DMF solvent (Merck) were used for preparation of the few layered MoS_2_ dispersion.

### Preparation of Go suspension

Graphite/sodium nitride powders (1:0.5 g) were added to 23 ml sulfuric acid followed by gradual adding of Potassium Permanganate (3 g). The obtained solution was magnetically stirred for 60 min at room temperature. A mixture of distilled water/hydrogen peroxide (45:170 ml) was then added to the solution and the final sediment (graphite oxide) was washed by hydrogen chloride and distilled water several times. Then, 0.1 g of the dried sediment powder was dispersed in 20 ml ethanol and subjected to a bath sonication system for 60 min to exfoliate graphite oxide flakes into the individual graphene oxide (Go) sheets.

### Preparation of few layered MoS_2_ suspension

A mixture was made from MoS_2_ powder (0.2 g) and DMF solvent (30 ml). Then, a probe sonication process (300 W, 8 s on, and 2 s off) was performed for 60 min at an ice bath followed by centrifugation at 2000 rpm to sediment the unexfoliated flakes. The MoS_2_ dispersion was obtained by collecting the top supernatant.

### Growth of vertically aligned CNTs

vertically aligned CNTs were grown using a plasma enhanced chemical vapor deposition (PECVD) system. Initially, a 285 nm thick oxide layer was thermally grown on a silicon wafer. A nickel layer with 10 nm thick was deposited on the oxide substrate by an electron beam evaporation technique and patterned into source/drain electrodes by a standard photo-lithography process. Nickel layer serves as a catalyst in the growth process of VACNTs. In the next step, the sample was placed in a quartz chamber followed by heating up to 700 ºC in a hydrogen atmosphere. At 700 ºC, the Ni film was exposed to the H_2_ plasma with power density of 5 W/cm^2^ for 10 min to convert the film into the nano islands. The sample was then exposed to a plasma mixture of hydrogen/acetylene (with ratio of 10:1) at plasma power density of 6.5 W/cm^2^ and pressure of 100 mtorr for 30 min. Then, a post-treatment process was applied by irradiating the sample to a H_2_ plasma at a low power density of 1.5 W/cm^2^ for 2 min in order to form C–H bonds on the surface of VACNTs at 700 ºC. The post-treatment step hydrogenated the dangling bonds of the nanotubes, made them unavailable for the gas molecules, and prevented them from being saturated during the gas response process.

### Device fabrication

10 nm nickel film was deposited on 285 nm SiO_2_/Si wafers using an electron beam evaporation system. The deposited film was patterned into interdigital electrodes by a photolithography approach followed by a dc-PECVD growth of the VACNTs. Next, a few droplets of the Go solution were drop-cast on the samples. In order to achieve rGo layer with an acceptable electrical conductivity, the prepared samples were placed in a quartz chamber, heated up to 600 ºC (heating rate of 15 ºC/min, under argon atmosphere at flow rate of 12 sccm, and chamber pressure of 1 torr), held for 60 min, and cooled down to room temperature under the same condition. Finally, MoS_2_ dispersion was drop cast onto the rGo/AVCNTs/SiO_2_/Si electrodes and dried in a vacuum at 110 ºC for 2 h.

### Characterizations

The AFM analyses was done by an NT-MDT microscope in non-contact modes. TEM images were taken by a Philips CM30 microscope at an operating voltage of 150 kV. SEM analyses was obtained by a SEM, Hitachi, S-4160 microscope. Raman spectra were measured by Senterra Raman system with a laser source of 532 nm. The electrical characterizations were done by a Keithly K361 source measure unit.

## Supplementary information

Supplementary file1 (DOCX 6352 kb)
