# Supplementary material for: Vertically aligned carbon nanotubes, MoS2–rGo based optoelectronic hybrids for NO2 gas sensing
Source: Sci Rep. 2020 Jul 9;10:11306. doi: 10.1038/s41598-020-68388-2 (PMC7347834; doi:10.1038/s41598-020-68388-2)
Supplement: Supplementary file 1 — Supplementary file1 (DOCX 6352 kb) [file 41598_2020_68388_MOESM1_ESM.docx]

Supporting Information

**Vertically Aligned Carbon Nanotubes, MoS_2_-rGo Based Optoelectronic Hybrids for NO_2_ gas sensing**

Foad Ghasemi

Nanoscale Physics Device Lab (NPDL), Department of Physics,

University of Kurdistan, Sanandaj, Iran, 66177-15175.

F.Ghasemi@uok.ac.ir


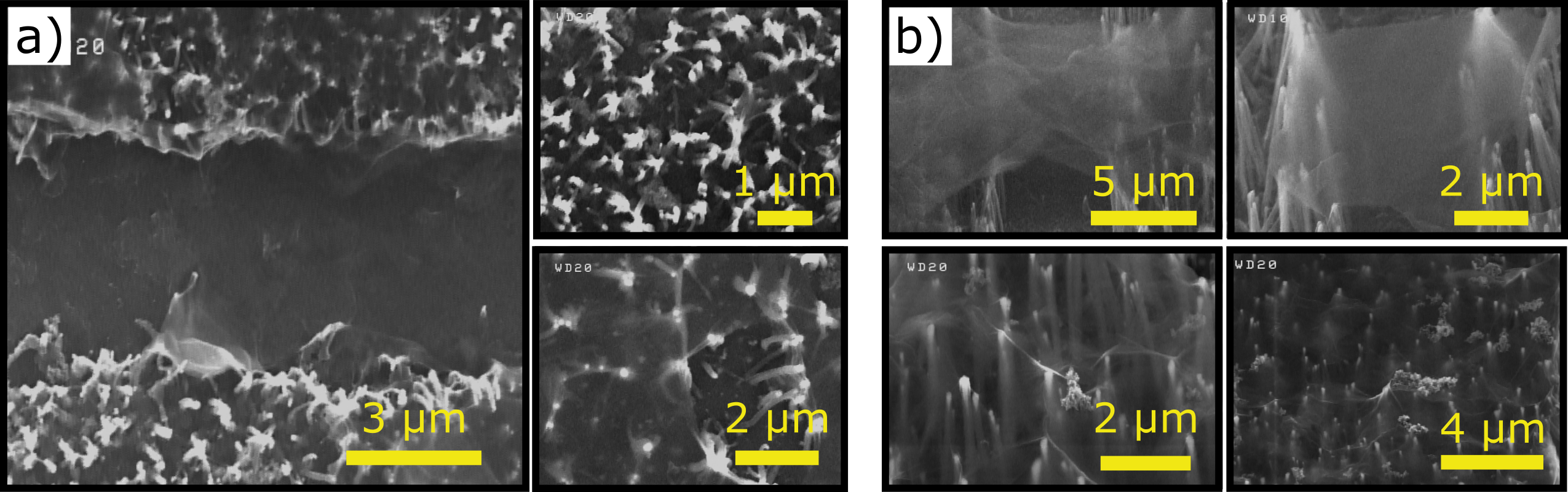


**Figure S1.** Device preparation through drop casting of Go solution from **a)** water solvent with lower sonication time. **b)** Ethanol solvent with higher sonication time.


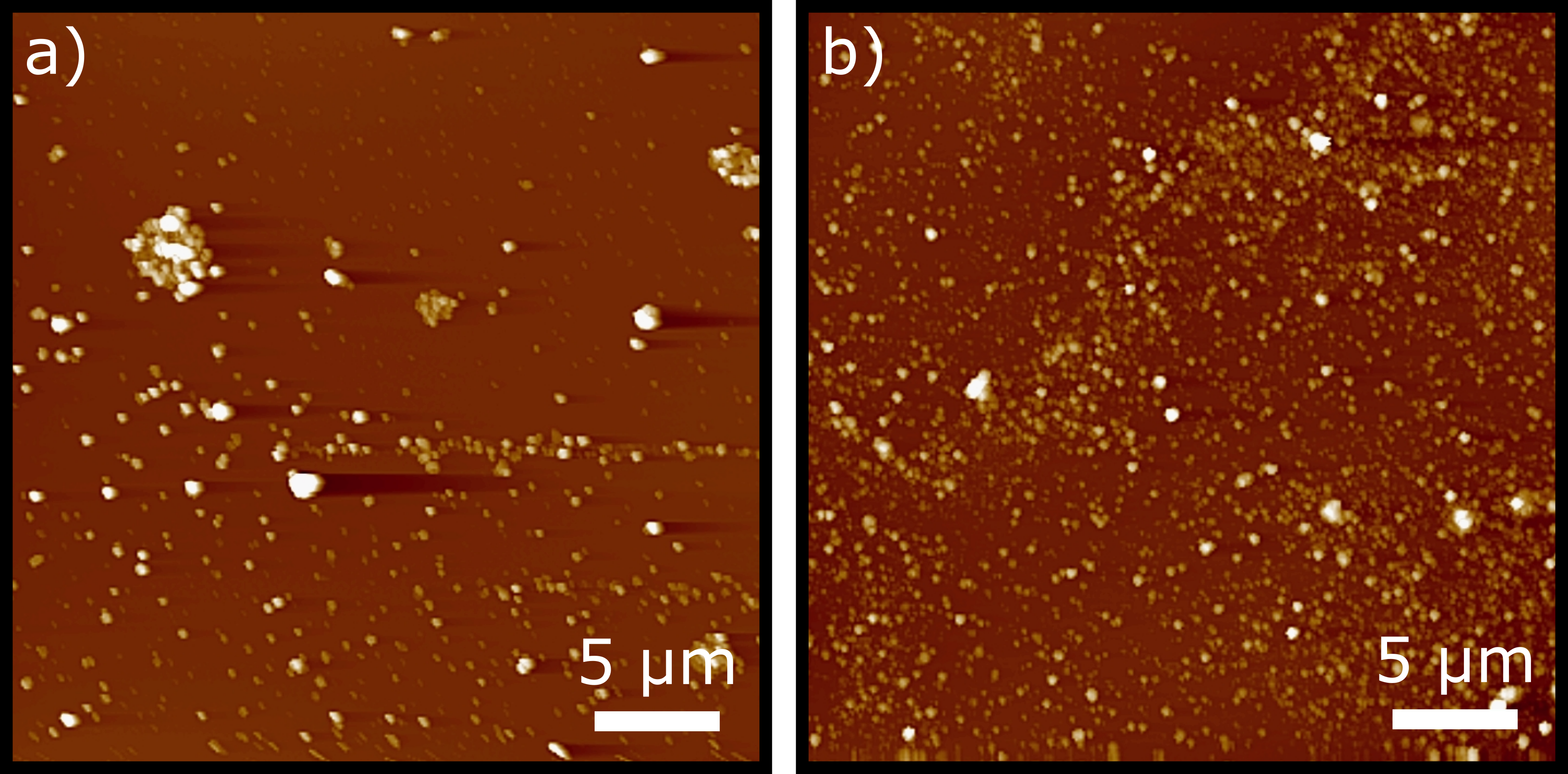


**Figure S2.** The effect of evaporation temperature on the deposited MoS_2_ flakes distribution over substrate in the drop casting process. **a)** Evaporation at 70 ˚C and **b)** evaporation at room temperature.
